# Andrographolide impedes cancer stemness and enhances radio-sensitivity in oral carcinomas via miR-218 activation

**DOI:** 10.18632/oncotarget.13755

**Published:** 2016-12-01

**Authors:** Po-Yu Yang, Pei-Ling Hsieh, Tong Hong Wang, Cheng-Chia Yu, Ming-Yi Lu, Yi-Wen Liao, Tzu-Hsin Lee, Chih-Yu Peng

**Affiliations:** ^1^ School of Dentistry, Chung Shan Medical University, Taichung, Taiwan; ^2^ Department of Dentistry, Chung Shan Medical University Hospital, Taichung, Taiwan; ^3^ Institute of Oral Sciences, Chung Shan Medical University, Taichung, Taiwan; ^4^ Oral Medicine Center, Chung Shan Medical University, Taichung, Taiwan; ^5^ Tissue Bank, Chang Gung Memorial Hospital, Tao-Yuan, Taiwan; ^6^ Research Center for Industry of Human Ecology, Chang Gung University of Science and Technology, Tao-Yuan, Taiwan; ^7^ Graduate Institute of Health Industry Technology, Chang Gung University of Science and Technology, Tao-Yuan, Taiwan

**Keywords:** oral squamous cell carcinomas, andrographolide, miR-218, cancer stemness

## Abstract

Current evidence suggests that oral cancer stem cells (OCSCs) possess high tumorigenic and metastatic properties as well as chemo- and radioresistance. In this study, we demonstrated that andrographolide, the main bioactive component in the medicinal plant Andrographis, significantly reduced oncogenicity and restored radio-sensitivity of ALDH1^+^CD44^+^ OCSCs. Mechanistic studies showed that andrographolide treatment increased the expression of microRNA-218 (miR-218), leading to the downregulation of Bmi1. We showed that knockdown of miR-218 in ALDH1^−^CD44^−^ non-OCSCs enhanced cancer stemness, while silencing of Bmi1 significantly counteracted it. Furthermore, we found tumor growth was reduced in mice bearing xenograft tumors after andrographolide treatment via activation of miR-218/Bmi1 axis. Together, these data demonstrated that the inhibition of tumor aggressiveness in OCSCs by andrographolide was mediated through the upregulation of miR-218, thereby reducing Bmi1 expression. These findings suggest that andrographolide may be a valuable natural compound for anti-CSCs treatment of OSCC.

## INTRODUCTION

Oral squamous cell carcinomas (OSCC) ranks as the sixth most common cancer with high incidence and represents a significant contributor to burden of cancer globally [1]. Unfortunately, treatments including extensive surgery, radiotherapy, chemotherapy or concurrent chemo/radiation are not effective for patients with advanced OSCC due to tumor recurrence, metastasis, or poor response to chemo/ radiotherapy [1]. It has been indicated that cervical lymph node metastasis is the major cause of death in OSCC patients [1]. As such, it is necessary to identify effective therapies for these patients and understand the molecular mechanisms underlying lymph node metastasis. Given that cancer stem cells (CSCs) have been considered to be responsible for metastasis and resistance to chemo/radiotherapy in OSCC [2–4], CSCs-targeted therapy may be a useful strategy against OSCC. Our previous study has shown that OSCC-CSCs were highly tumorigenic, metastatic, resistant to radio/chemotherapy and had increased expression of epithelial-mesenchymal transition (EMT) markers [3]. Other recent reports revealed that CD44 [5], CD133 [6], aldehyde dehydrogenase (ALDH) [7], membrane GRP78 [8], side population [9] and c-Met [10] could be used to detect CSCs from OSCC as well.

*Andrographis paniculata* (Burm. f) Ness is an herbal plant in the Acanthaceae family. It is widely cultivated in India, Thailand and China and has been used as a traditional medicine to treat various diseases [11]. The most abundant diterpene lactone in the leaves and stem is called andrographolide and it exhibits numerous bioactive properties, including anti-cancer [12], anti-inflammation [13], hepatoprotection [14] and anti-infection [15]. Several studies have demonstrated its anti-cancer potential and possible mechanisms. It has been reported that andrographolide reduces invasiveness of human non-small cell lung cancer A549 cells by inhibiting MMP-7 expression through downregulation of PI3K/Akt signaling pathway [16]. It is able to cause cell growth suppression of human colorectal carcinoma LoVo cells by promoting cell-cycle arrest at G_1_/S phase and increasing the expression of p53, p21 and p16 [17]. And it has been shown to inhibit NF-κB-induced bcl-2 activation and modulate p53-mediated caspase-3 gene expression, thereby increasing apoptosis of B16F-10 melanoma cells [18]. Andrographolide is also a potent inhibitor for multiple myeloma [19] and melanoma CSCs [20]. Nevertheless, efficacy of using andrographolide in the specific subset of OCSCs still remains to be determined.

MicroRNAs (miRNAs), a class of small noncoding RNAs regulating the gene expression at the post-transcriptional level by binding to target mRNAs within the 3′ untranslated region (UTR), have been found to be involved in many important biological processes [21–23]. In particular, it has been found that miR-218 acts as a tumor suppressor by targeting many oncogenes related to proliferation [24], apoptosis [25] and invasion [26]. miR-218 is a vertebrate-specific intronic miRNA coexpressed with its host genes, tumor suppressor gene SLIT2/3. The mature form of miR-218 is generated from two separate loci, miR-218-1 and miR-218-2, which are located on chromosomes 4p15.31 and 5q35.1 within the introns of SLIT2 and SLIT3, respectively [27]. Several studies have shown that miR-218 is significantly downregulated in colorectal cancer [28], breast cancer [29], clear cell renal cell carcinoma [30], supporting miR-218 as a key factor in human tumorigenesis. Also, it has been linked to the regulation of cancer stemness. For example, miR-218 attenuates self-renewal in glioma stem-like cells [31]. Most importantly, previous study showed that miR-218 expression is significantly upregulated by Andro pretreatment in human alveolar epithelial A549 cells with reduced inflammatory response and oxidative stress [32]. Consequently, it is imperative to elucidate the relationship between Andro and miR-218 in eliciting anti-OSCC activity.

In the current study, we evaluated the effect of andrographolide on cell survival, self-renewal, expression of cancer stem cell markers *in vitro* and tumorigenecity *in vivo*. We also found administration of andrographolide enhanced the tumor sensitization to radiation therapy. In addition, our results indicated that miR-218 may play a pivotal role in the anti-CSCs property of andrographolide by targeting Bmi1. Overall, we demonstrated the tumor suppressive activity of andrographolide in OCSC and provided evidence for downstream mechanisms involved in this anti-cancer response.

## RESULTS

### Andrographolide reduces cell proliferation and self-renewal in oral cancer stem cells (OCSCs)

**Figure 1:**
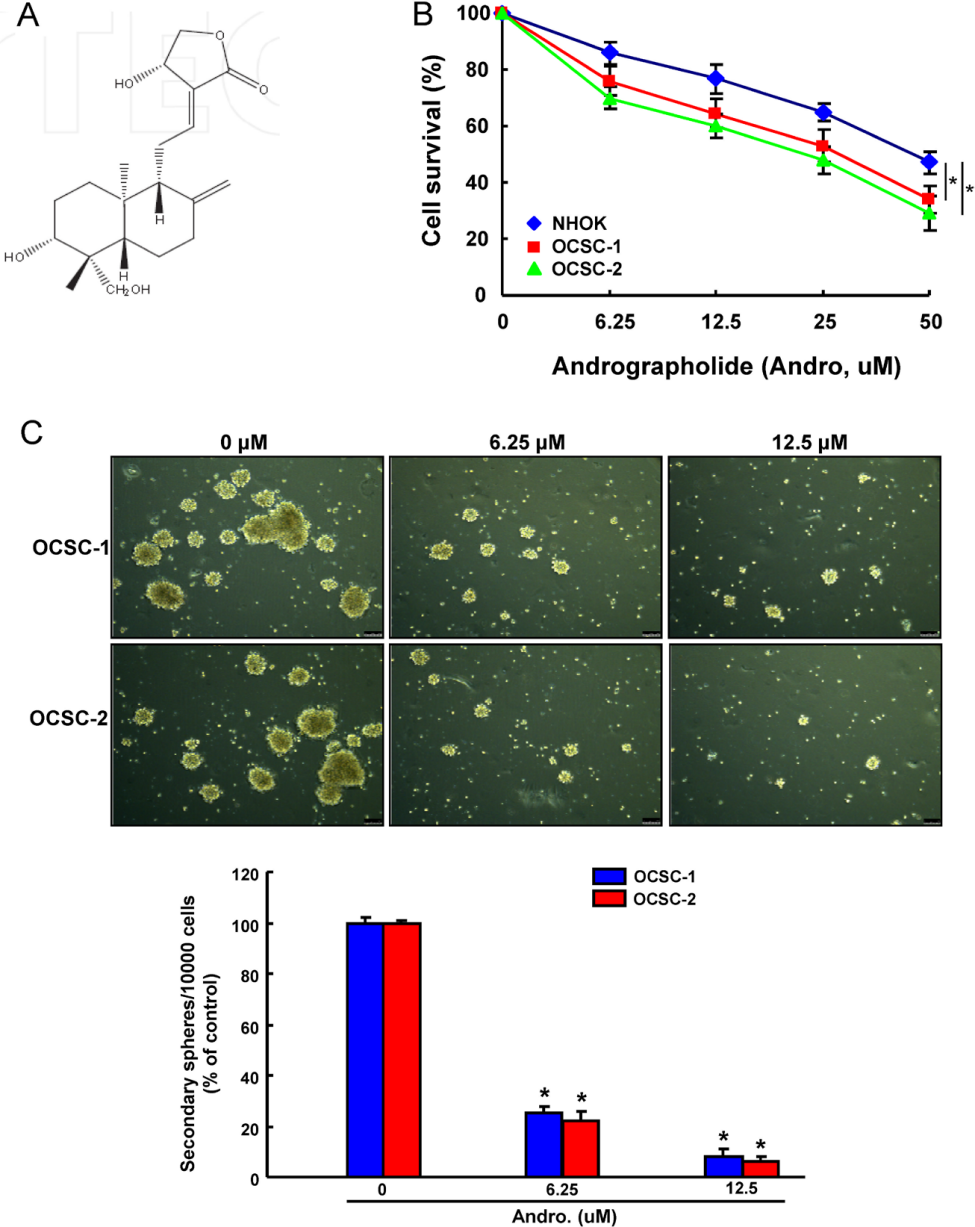
The cytotoxicity and effect of andrographolide on OCSCs self-renewal property (**A**)The chemical structure of andrographolide; (**B**) NHOK cells and two ALDH^+^CD44^+^ OCSCs from primary cultivated OSCC were treated with various concentrations of andrographolide for 24 hr followed by MTT assay; (**C**) OCSCs treated with or without andrographolide were subjected to a secondary sphere-forming assay. The number of spheres was calculated and data was presented as percentage of control. Experiments were performed in triplicate. Values are expressed as mean ± SD. *p < .05 compared to control.

ALDH1^+^CD44^+^ OCSCs were isolated from patient-derived cell lines and used to study the CSC properties, since ALDH1 and CD44 have been recognized as markers to distinguish malignant from premalignant cells as well as identify the putative OCSCs [33, 34]. We first investigated the cell survival of these two ALDH1^+^CD44^+^ OCSCs and normal human oral keratinocytes (NHOK) in order to understand the cytotoxicity of Andrographolide (Figure 1A). As shown in Figure 1B, andrographolide markedly suppressed the viability of two OCSCs in a dose-dependent manner using MTT assay.

Successful sphere formation following serial passages of culture is one of the characteristics to access the capacity of persistent self-renewal in CSCs [35]. Therefore, we evaluated the secondary sphere-forming ability of OCSCs after andrographolide treatment. Our result demonstrated that andrographolide exhibited a strong anti-sphere forming potential in a dose-dependent fashion (Figure 1C) and effectively inhibited their capacity of self-renewal in OCSCs.

### Andrographolide represses ALDH1 activity and the expression of stemness signatures in OCSCs

**Figure 2:**
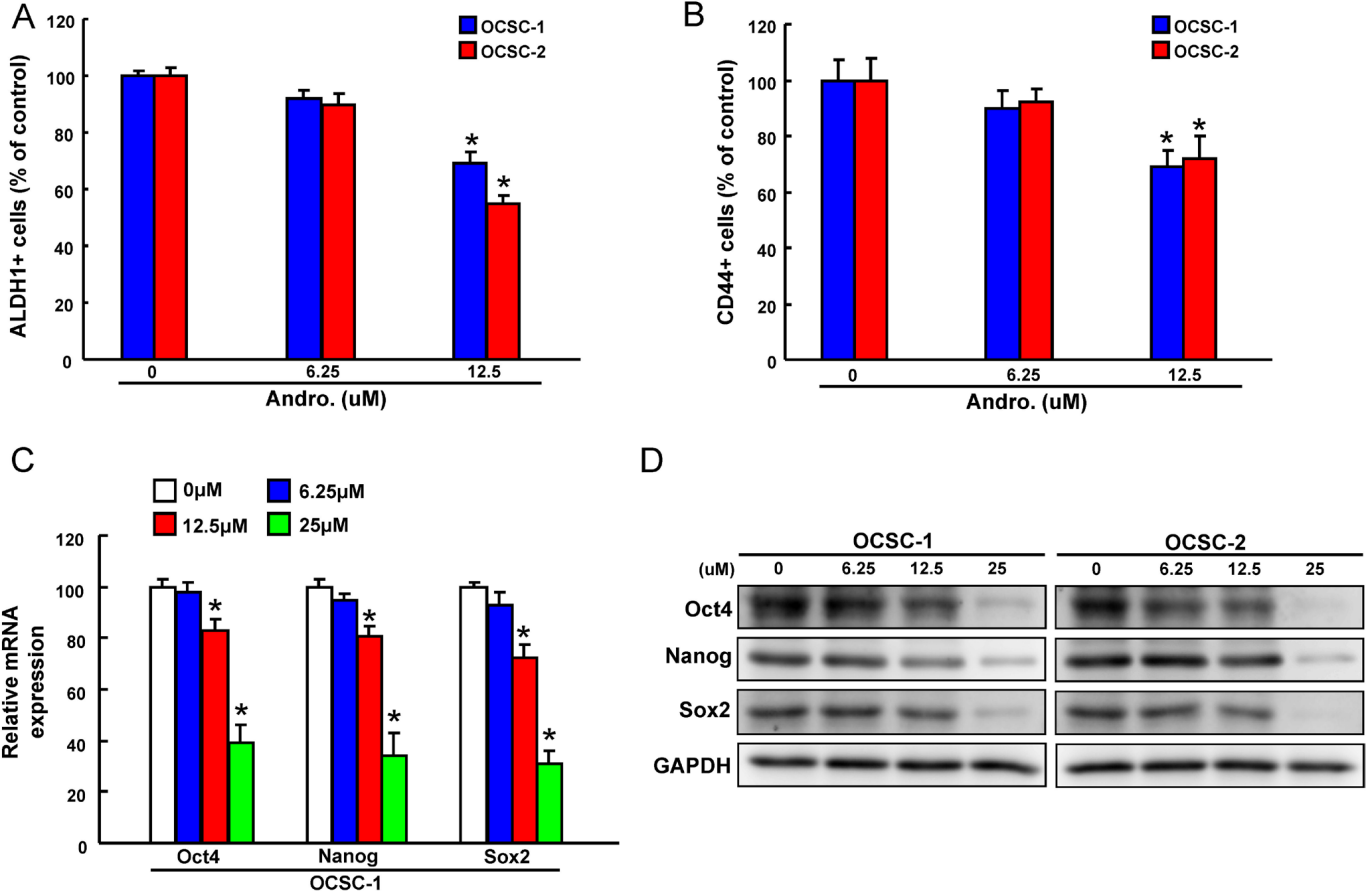
The effect of andrographolide on cancer stemness marker expression (**A**)The ALDH1 activity and (**B**) CD44 positivity of OCSCs treated with or without andrographolide was assessed by flow cytometry and data was presented as percentage of control; (B) mRNA and (**C**) protein expressions of the stemness markers (Oct4, Nanog, and Sox2) in andrographolide-treated OCSCs. Experiments were performed in triplicate. Values are expressed as mean ± SD. *p < .05 compared to control.

ALDH1 enzymatic activity was detected by ALDEFLUOR assay and our data suggested administration of andrographolide significantly resulted in a concentration-dependent decrease in ALDH1 activity of both OCSCs (Figure 2A). Andrographolide treatment also suppressed the percentages of CD44^+^ cells (Figure 2B). To further determine whether the reduction in CSCs hallmarks following andrographolide treatment was associated with the expression of stemness-related markers, the levels of Oct-4, Nanog and Sox2 in OCSCs were analyzed by real-time PCR and western blot. The expression of mRNA (Figure 2C) and protein (Figure 2D) levels of Oct-4, Nanog, and Sox2 in both OCSCs were significantly reduced.

### Inhibition of oncogenicity and enhanced radio-sensitivity in OCSCs by Andrographolide

**Figure 3:**
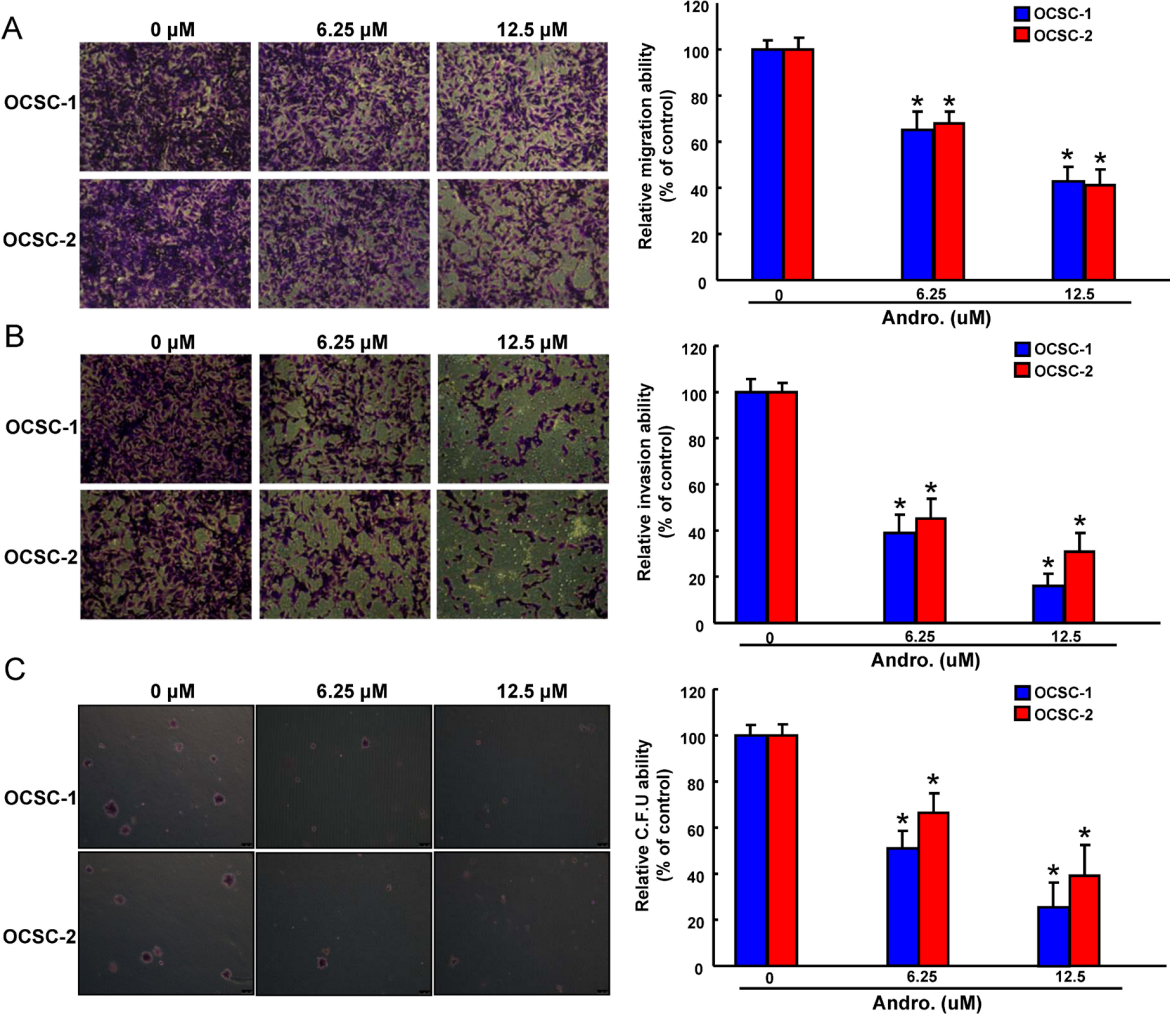
Anti-oncogenic effect of andrographolide in OCSCs Representative images (left) and quantification (right) of (**A**) migration assay, (**B**) Matrigel invasion assay and (**C**) soft agar colony formation assay of OCSCs treated with various concentration of andrographolide. Experiments were performed in triplicate. Values are expressed as mean ± SD. *p < .05 compared to control.

Since CSCs appear to play a critical role in tumorigenesis and metastasis [36], it is extremely important to assess oncogenicity of OCSCs when testing the efficacy of andrographolide. Overall, a dose-dependent suppression of tumor-initiating activity including migration (Figure 3A), invasion (Figure 3B), and colony formation (Figure 3C) was observed. There results clearly demonstrated that andrographolide exerted a pronounced oncogenic–inhibitory effect.

**Figure 4:**
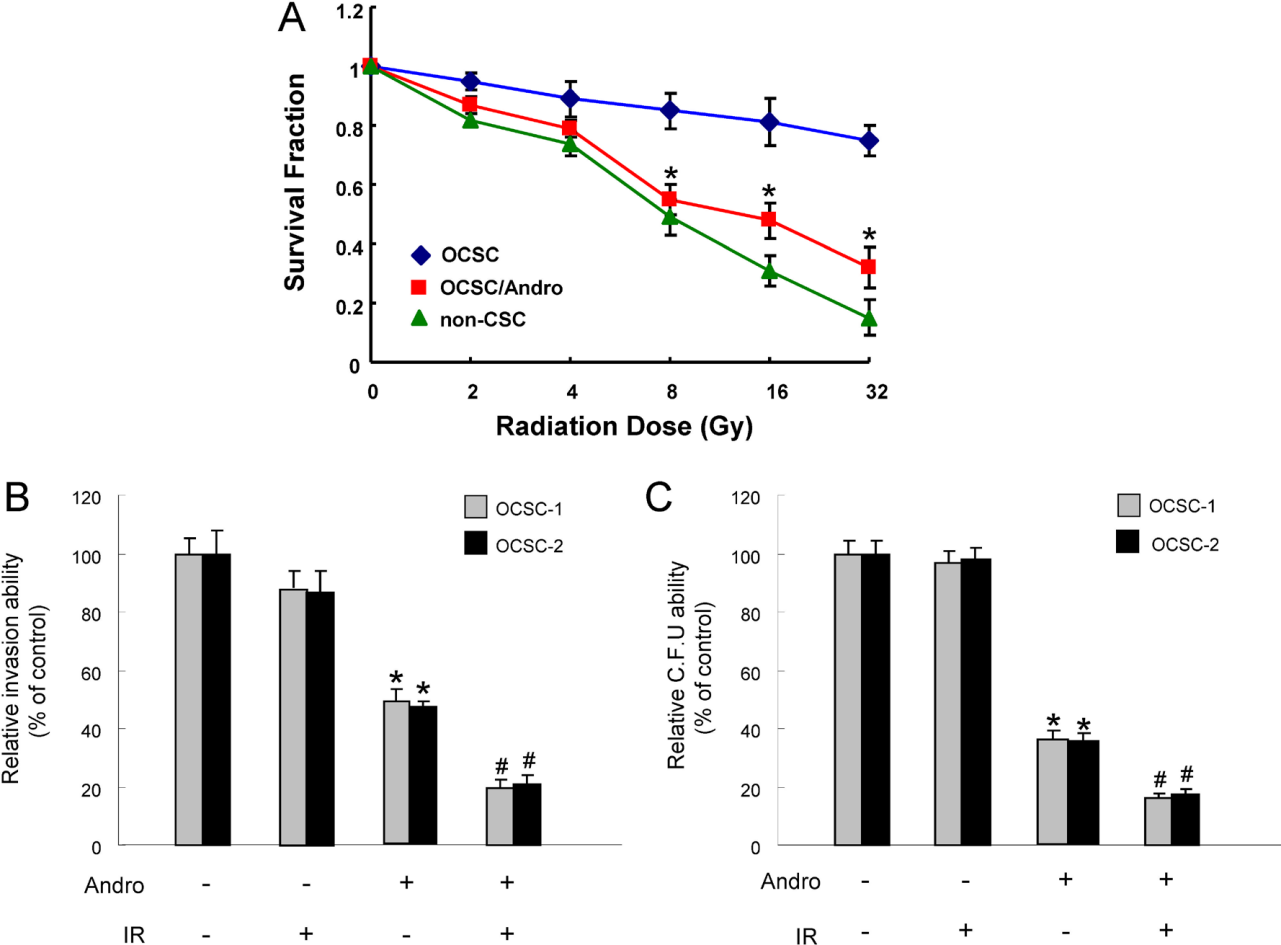
The effect of andrographolide on radio-sensitivity of OCSC (**A**)The surviving fractions of cancer cells or andrographolide-treated OCSCs were evaluated after various doses of radiation exposure; (**B**) Invasion and (**C**) colony-forming ability in OCSCs were examined after treatment with either andrographolide/radiotherapy alone or combination of both. *p < .05 andrographolide alone vs. control; #*p* < .05 andrographolide+ IR vs. andrographolide alone.

Reemergence of radiotherapy-resistant CSCs is considered to be responsible for the recurrence of cancers after conventional therapeutic treatments [37]. As expected, OCSCs were more radio-resistant compared with the parental OSCC using cell viability assay. Nevertheless, the sensitivity to radiation therapy in OCSCs was dramatically improved in combination with andrographolide (Figure 4A). We also observed an enhanced antitumor activity via the synergic action of andrographolide and radiation in invasion (Figure 4B) and colony-forming potentials (Figure 4C) of OCSCs.

### Andrographolide increases the expression of tumor suppressive miR-218

**Figure 5:**
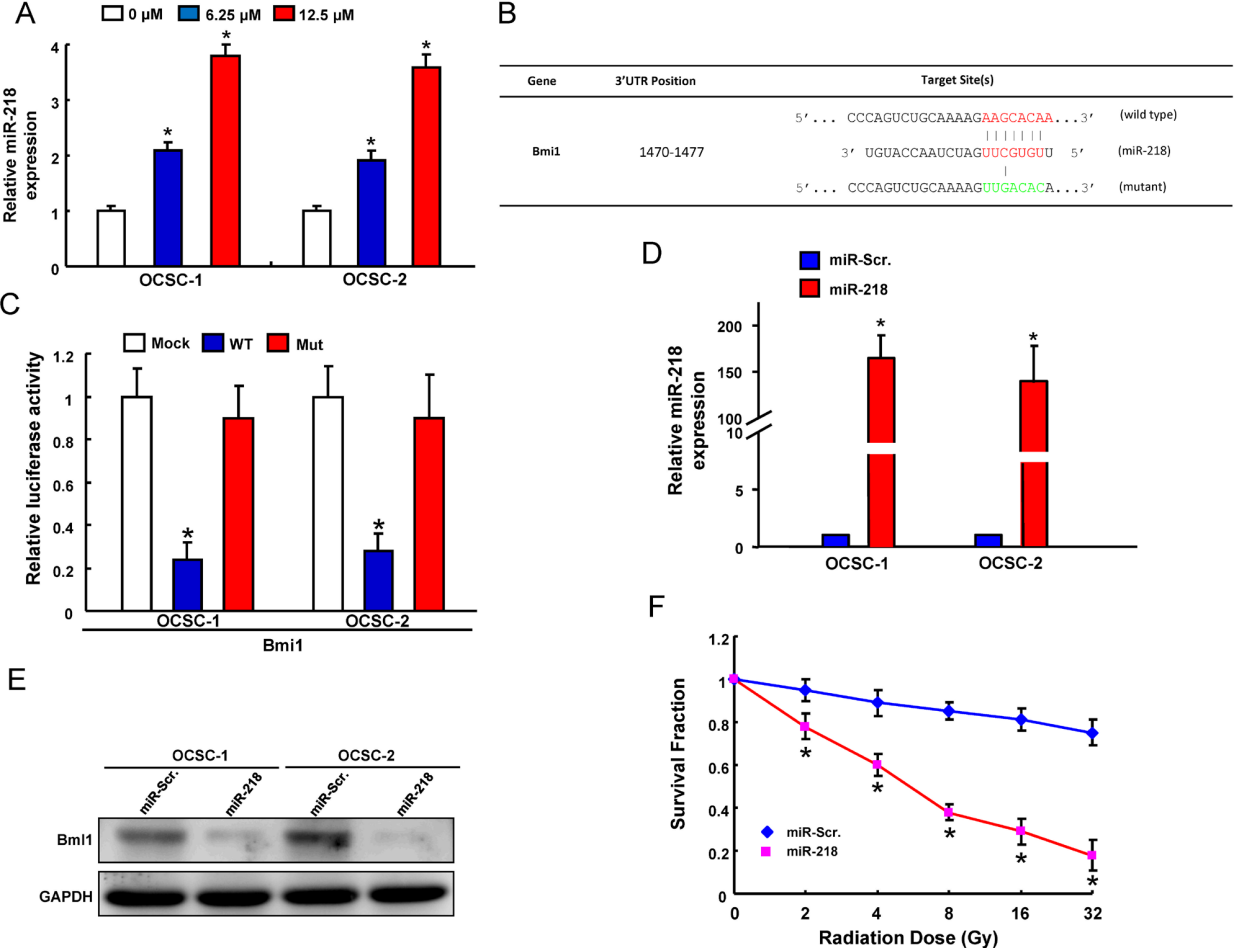
Identification of Bmi18 as a direct target of miR-218 in OCSCs (**A**) miR-218 expression in andrographolide-treated OCSCs; (**B**) Schematic representation of the constructed Bmi1 3′UTR reporter plasmids; (**C**) The wild-type or mutant form of Bmi1 reporter was co-transfected with miR-218 or empty vector (Mock) into OCSCs. The luciferase activity was assessed and presented as relative units to Mock-treated cells; (**D**) mRNA expression of miR-218 and (**E**) protein expression of Bmi1 in OCSCs transfected with pLV-miR-scrambled (pLV-miR-Scr.) or pLV-miR-218; (**F**) Survival fraction of pLV-miR-Scr or pLV-miR-218-treated OCSCs after various doses of radiation. Experiments were performed in triplicate. Values are expressed as mean ± SD. *p < .05 compared to control.

Numerous studies have implicated miRNAs involve in the regulation of CSCs properties [38–40]. In the current study, we found delivery of andrographolide caused a dose-dependent increase in the level of miR-218 expression using real-time RT-PCR analysis (Figure 5A). Following identification of Bmi1 as the potential target of miR-218 through Target Scan program, we constructed reporter plasmids containing either full-length or mutated forms of the 3′UTR region of Bmi1 (Figure 5B). Luciferase reporter assay showed that miR-218 decreased the luciferase activity of reporter plasmids containing full-length Bmi1 3′UTR and this phenomenon was not observed in mutant form (Figure 5C), confirming Bmi1 as a target of miR-218 in OCSCs. Furthermore, we exogenously overexpressed miR-218 in OCSCs and the efficiency was validated by real-time RT-PCR analysis (Figure 5D). In accordance with the previous finding, the protein level of Bmi1 was decreased in the miR-218-overexpressing OCSCs (Figure 5E). Most importantly, overexpression of miR-218 significantly suppressed the radio-resistance (Figure 5F). Overexpression of miR-218 significantly suppressed self-renewal ability (Supplementary Figure S1A) and nvasion capacity (Supplementary Figure S1B) in OCSCs.

### miR-218 suppresses cancer stemness and invasiveness by targeting Bmi1

**Figure 6:**
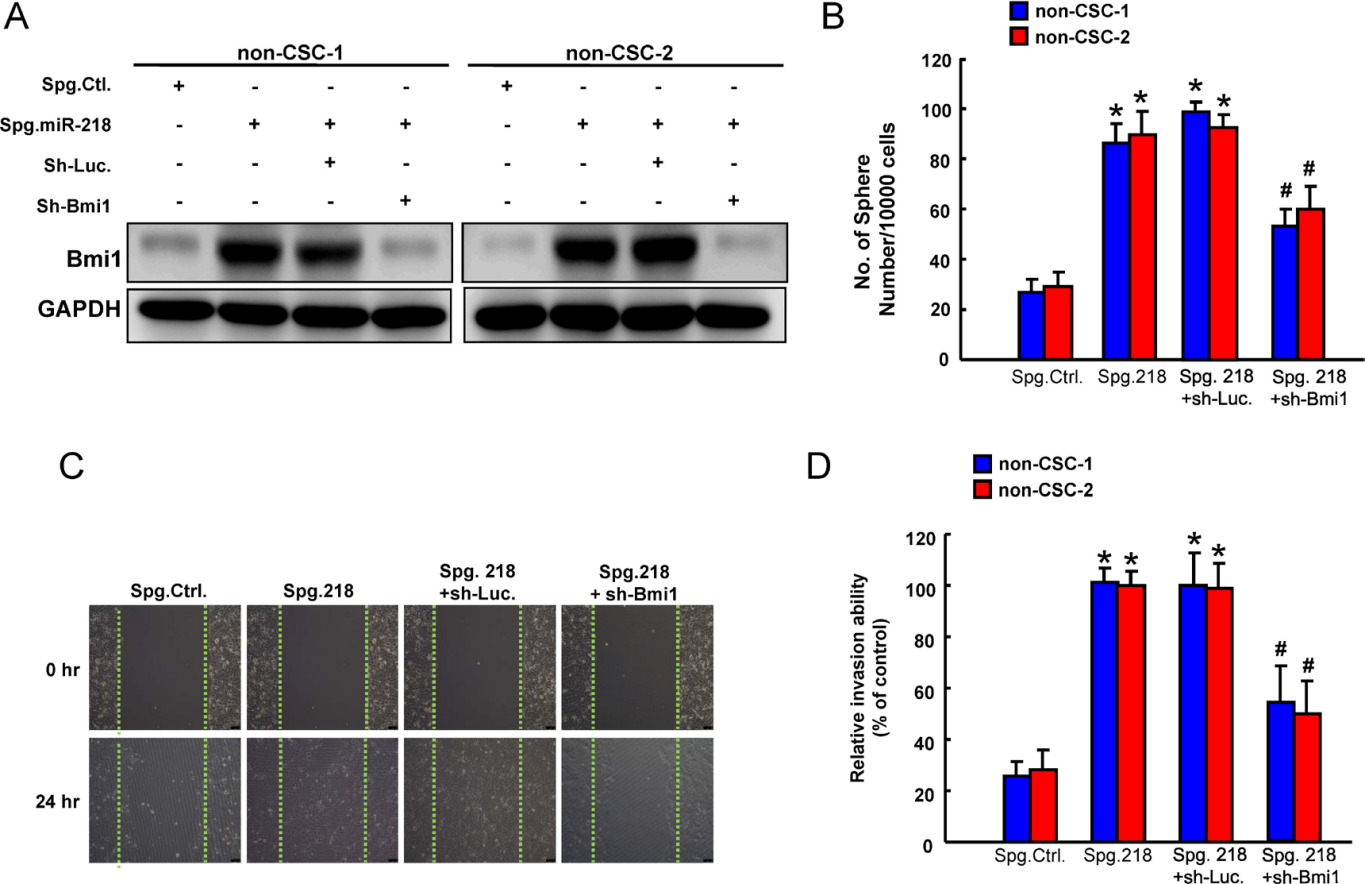
Involvement of Bmi1 in tumor suppressive function of miR-218 (**A**) Protein expression of Bmi1, (**B**) sphere formation, (**C**) wound-healing and (**D**) invasion ability in ALDH1^−^CD44^−^ non cancer stem cells (non-CSC) transfected with Spg-Ctl, Spg-miR-218, sh-Luc or sh-Bmi1. Experiments were performed in triplicate. Values are expressed as mean ± SD. *p < .05 compared to Spg.Ctrl.;# *p* < .05 Spg. 218+sh-Bmi1 compared to Spg. 218+sh-Luc.

The role of Bmi1 in the miR-218-mediated inhibition of cancer stemness was further clarified. First, the elevated expression of Bmi1 in non-CSCs treated with miR-218 knockdown sponge (Spg-miR-218) was verified by western blotting (Figure 6A). We showed that silencing of endogenous miR-218 induced sphere-forming capability in non-CSCs, which was abolished by knockdown of Bmi1 (Figure 6B). Also, wound-healing (Figure 6C) and invasion (Figure 6D) abilities were increased in Spg-miR-218-treated non-CSCs, and silencing of Bmi1 counteracted these responses (Figure 6C–6D). Taken together, these results demonstrated that miR-218-induced downregulation of Bmi1 suppressed self-renewal, tumor cell motility and invasion in OSCC cells.

### Administration of andrographolide exerts an inhibitory effect on tumor growth *in vivo* through miR-218 activation

**Figure 7:**
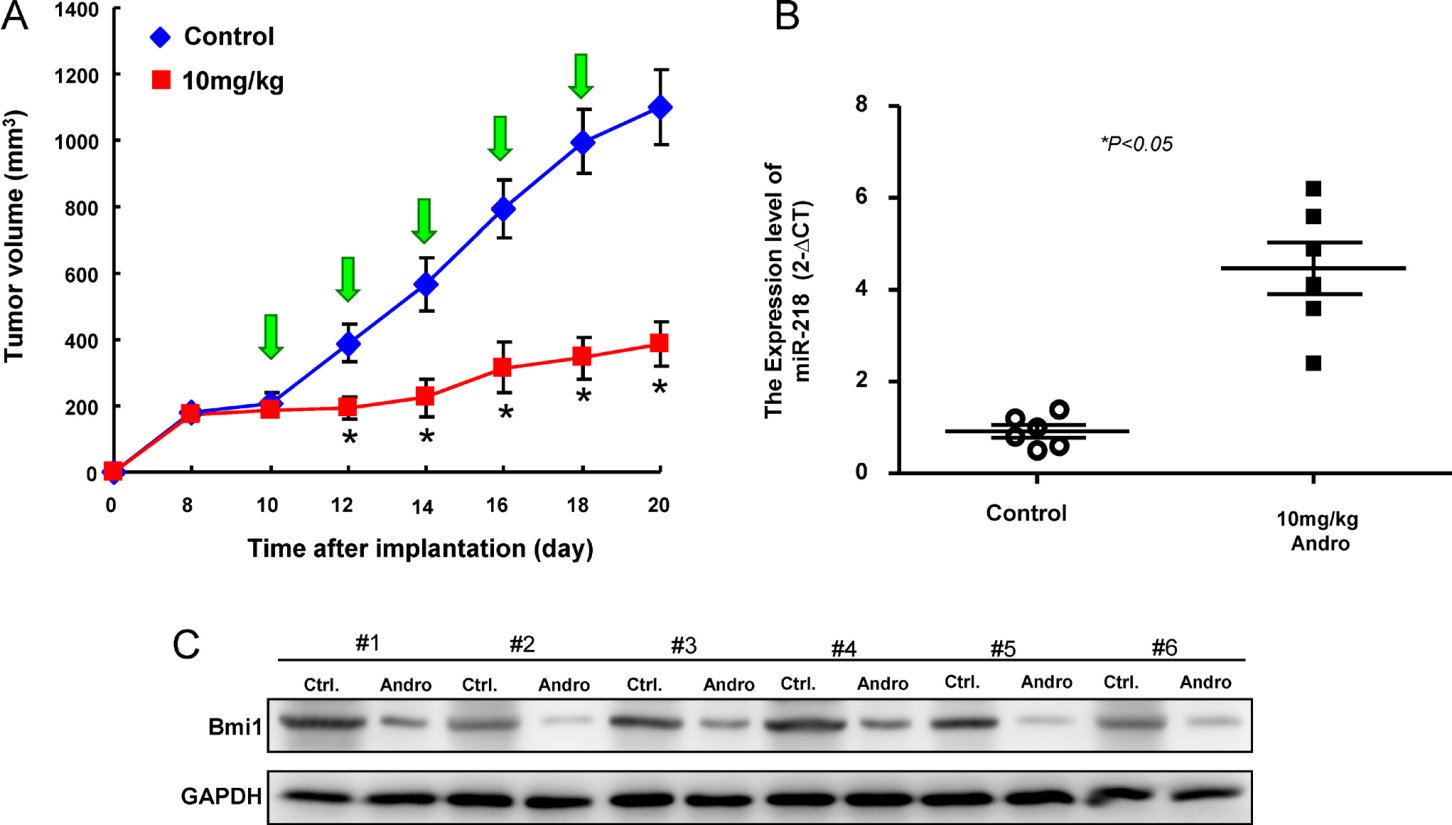
Inhibition of tumor growth by Andrographolide via miR-21/Bmi1 axis (**A**) Quantification of tumor volume changes following administration of Andrographolide; (**B**) relative miR-218 expression and (**C**) Bmi1 expression in the excised tumors. Experiments were performed in triplicate. Values are expressed as mean ± SD. *p < .05 compared to control.

To validate the efficacy of andrographolide-mediated anti-tumorigenic function *in vivo*, immunocompromised mice bearing OCSC xenografts received andrographolide treatment or vehicle (water) by oral gavage. We found that tumor formation was significantly suppressed following administration of andrographolide by day 20 compared to vehicle group (Figure 7A). The upregulation of miR-218 (Figure 7B) and downregulation of Bmi1 (Figure 7C) were confirmed by real-time RT-PCR and western blotting analysis, respectively.

## DISCUSSION

Metastasis is the main reason for the high mortality of cancer and cervical lymph node metastasis has been considered to be the most important adverse prognostic factor and major cause of death for OSCC patients [1]. Among various factors regulating metastasis properties, miR-218 has been recently identified as a metastasis suppressor. It was found that miR-218 was dramatically downregulated in metastatic prostate [41] and cervical cancer cells [32]. Overexpression of miR-218 was shown to inhibit lung cancer metastasis in tumor bearing mice [32, 42]. Additionally, several studies have reported that suppression of miR-218 resulted in increased invasive ability of gastric cancer [43], glioma [44] or pancreatic ductal adenocarcinoma cells [45], whereas restoration of miR-218 led to significant inhibition of cell migration and invasion in OSCC [46], cervical squamous cell carcinoma [42] and renal carcinoma cells [47]. Consistent with the previous studies, we showed knockdown of miR-218 expression in non-CSCs induced self-renewal, tumor cell motility and invasion in OSCC, while knockdown of Bmi1 effectively reversed these phenomena (Figure 5). To our knowledge, this is the first report demonstrating miR-218 expression is crucial for maintaining metastasis of OSCC via targeting Bmi1.

B-cell-specific Moloney murine leukemia virus integration site 1 (Bmi1) is a member of polycomb repressive complex 1, and it has been implicated as one of the important regulators of self-renewal in stem cells [48, 49]. On other hand, epithelial-mesenchymal transition (EMT), a critical process involved in the transdifferentiation of polarized epithelial cells into an invasive mesenchymal phenotype, has emerged as an essential driver of malignant transformation including tumorigenicity, metastasis and cancer stemness [50]. It has been indicated that Bmi1 acts synergistically with Twist to promote EMT and tumor-initiating capability in OSCC and the increased expression of Bmi1 is correlated with unfavourable prognosis [51]. Apart from induction of EMT and cancer cell stemness, growing evidence suggests that tumor recurrence and chemoresistance of CSCs are in part due to the activation of Bmi1 [52, 53]. Recent studies have revealed that downregulation of Bmi1 enhanced the sensitivity of chemotherapeutic drugs in various carcinomas by regulating oxidative stress and inducing apoptosis [54–56]. In the present study, we demonstrated the role of Bmi1 in the miR-218-mediated inhibition of cancer stemness and invasiveness. Future research should thoroughly explore the miR-218/Bmi1 axis in regulation of EMT in OSCC.

OSCC is often associated with high incidence of tumor recurrences, which account for the majority of treatment failures post-surgery or conventional radiotherapy. Cancer recurrence is partly attributable to radioresistance and represents a major clinical challenge. In order to obtain a better therapeutic outcome of malignant OSCC, it is necessary to develop more specific treatment options and effective radiosensitizers. Numerous studies have suggested that andrographolide enhanced radiosensitivity in human ovarian cancer SKOV3 xenografts [57] and esophageal cancer cells [58] through apoptosis induction as well as Ras-transformed cells to radiation both *in vitro* and *in vivo* [59]. In associated with these studies, we also found andrographolide combined with radiation displayed synergistic effect on invasiveness and clonogenicity, leading to significantly suppressed tumorigenesis in OCSCs.

In conclusion, our data indicate that andrographolide can inhibit oncogenic properties of OCSCs through activation of miR-218-targeting Bmi1. Furthermore, andrographolide has radiosensitizing effect and may serve as an adjuvant therapy to reduce the rate of recurrence. These findings provide a strong rationale for the potential use of andrographolide as a radiosensitizer and anti-CSCs agent.

## MATERIALS AND METHODS

### Reagents

Andrographolide was purchased from Sigma-Aldrich Chemical Co. (St. Louis, MO, USA) and dissolved in DMSO (Merck, Darmstadt, Germany) to obtain a stock solution of 100 mM. Andrographolide was further diluted in culture medium to the appropriate final concentrations prior to use.

### Isolation of oral cancer stem cells

All procedures of tissue acquirements were approved by Institutional Review Committee at Chung Shan Medical University. Primary oral cancer cell cultures were established as previously described [60, 61]. To identify ALDH1^+^CD44^+^ oral cancer stem cells, we stained cells with ALDEFLUOR assay kit (StemCell Technologies, Vancouver, BC, Canada) and anti-CD44 antibody conjugated to phycoerythrin (Miltenyi Biotech., Auburn, CA, USA) followed by fluorescence-activated cell sorting using FACSAria II cell sorter (BD Biosciences, San Jose, CA, USA).

### MTT assay

Cells were incubated with different concentration of andrographolide-containing medium or vehicle (0.1 % DMSO) at 37°C for 24 hr followed by MTT ((3-(4,5-dimethylthiazol-2-yl)-2,5-diphenyl tetrazolium bromide) treatment for 3 h. The blue formazan crystals of viable cells were dissolved in DMSO and then evaluated spectrophotometrically at 570 nm. DMSO-treated group was set as 100%, and data were presented as percentage of DMSO control.

### Tumorsphere-forming assay

Tumor cells were dissociated and cultured as tumorspheres in modified medium consisting of DMEM/F-12 supplemented with N2 (R&D Systems, Minneapolis, MN, USA), 10 ng/mL epidermal growth factor (EGF, Invitrogen, Carlsbad, CA, USA), 10 ng/mL basic fibroblast growth factor (bFGF, Invitrogen), and penicillin/streptomycin at 10^3^ live cells per well of ultra-low attachment six-well plate (Corning, NY, USA). Medium was changed every other day for two weeks. The number of tumor spheres formed were observed and counted under a microscope.

### ALDH1 activity assay

ALDH1 activity was examined using ALDEFLUOR assay kit (StemCell Technologies). 5 × 10^5^ cells were suspended in 1ml ALDEFLUOR assay buffer containing the ALDH substrate and incubated at 37°C for 60 minutes. ALDH^+^ cells were analyzed by flow cytometry (FACSCalibur; BD Biosciences) to compare the fluorescence intensity with the DEAB (diethylaminobenzaldehyde)-treated negative control.

### Quantitative real-time reverse-transcriptase (RT)-PCR

Total RNA was extracted from cells or tissues using Trizol reagent (Invitrogen). cDNA synthesis was performed using Superscript III first-strand synthesis system (Invitrogen) according to the manufacturer's instruction. GAPDH housekeeping gene was used as reference. Amplification and detection were carried out on an ABI StepOne™ Real-Time PCR Systems (Applied Biosystems, Carlsbad, CA, USA) for analysis of stemness-related markers. miR-218 level was quantified by TaqMan miRNA assays with specific primer sets (Applied Biosystems). All reagents and protocols were from Applied Biosystems, and detection was performed using 7900HT fast real-time PCR system.

### Western blot

Cells were lysed in NP-40 buffer and protein concentration was determined using BCA protein assay kit (Thermo Fisher Scientific, Rockford, IL, USA). Samples (25 μg of total protein) were separated by 10% SDS-PAGE and wet-transferred to a PVDF membrane (Millipore, Billerca, MA, USA). The membrane was incubated with primary antibodies recognizing Oct4 (#2750, Cell Signaling, Beverly, MA, USA), Nanog (#3850, Cell Signaling), Sox2 (#3579, Cell Signaling) or GAPDH (GTX627408, GeneTex, Irvine, CA, USA). Following primary antibodies, the membrane was incubated with corresponding secondary antibodies. The immunoreactive bands were developed using an ECL-plus chemiluminescence substrate (Perkin-Elmer, Waltham, MA, USA) and captured by LAS-1000 plus Luminescent Image Analyzer (GE Healthcare, Piscataway, NJ, USA).

### Migration/invasion assay

Cell migration and invasion assays were carried out using 24-well plate Transwell^®^ system with a polycarbonate filter membrane of 8-μm pore size (Corning). For invasion assay, the membrane was coated with Matrigel. The cells were seeded to the upper compartment at the density of 1 × 10^5^ in 250 μL serum-free medium and medium supplemented with 10% FBS was used as a chemoattractant in the lower chamber. After 24 h of incubation, the filter membrane was stained with 0.1% Crystal Violet. The cells were then visualized and counted from five different fields of 100-fold magnification under an inverted microscope.

### Soft agar colony forming assay

Each well of a six-well culture dish was coated with 1 ml of bottom agar (Sigma-Aldrich) mixture (DMEM/F-12, 15% (v/v) FBS, 0.525% (w/v) agar). After the bottom layer was solidified, 1 ml of top agar-medium mixture (DMEM/F-12, 15% (v/v) FCS, 0.3% (w/v) agar) containing 5 × 10^4^ cells was added, and the dishes were incubated at 37°C for 2 weeks. Plates were stained with 0.01% Crystal Violet, and then the colonies were counted.

### miR-218 Sponge

Oligos for miR-218 sponge, and scramble construction were constructed using a pcDNA 6.2-GW/EmGFP-miR plasmid (Invitrogen). MicroRNA SPONGE sequence design was based on previous report [41]. Further multiple copy amplifications were done with recovery of BamH1 and XhoI digested fragments and subcloned into pcDNA 6.2-GW/EmGFP-miR plasmid [3].

### *In vivo* imaging of tumor growth

All procedures involving animals were conducted in accordance with the institutional animal welfare guidelines of the Chung Shan Medical University. 5–6 weeks old immuno-deficient nude mice (BALB/c nu/nu mice) were used for the xenograft model. OCSC (1 × 10^4^ cells/0.2 mL/mouse) were injected subcutaneously into the right axilla and the day of cell implantation was designated day 0. The mice were randomly divided into two groups and fed with either saline (control) or andrographolide (10 mg/day/kg) by oral gavage 10 days post implantation. Tumor size measurement was performed using an IVIS50 animal imaging system (Xenogen Corp.). The volume was calculated (according to the following formula: [length × width^2^]/2), and then analyzed by Image-Pro Plus software. Body weight was assessed daily after cell injection. After 20 days, the animals were euthanized, and the primary tumors were weighed and for miR-218/Bmi1 analysis.

### Statistical analysis

Statistical Package of Social Sciences software (SPSS; version 13.0) was used for statistical analysis. Student's *t* test or ANOVA analysis were used to determine statistical significance of the differences between experimental groups; *p* values less than 0.05 were considered statistically significant.

## SUPPLEMENTARY MATERIALS FIGURES


