# Supplementary material for: Andrographolide impedes cancer stemness and enhances radio-sensitivity in oral carcinomas via miR-218 activation
Source: Oncotarget. 2016 Dec 1;8(3):4196–207. doi: 10.18632/oncotarget.13755 (PMC5354823; doi:10.18632/oncotarget.13755)
Supplement: Supplementary file 1 [file oncotarget-08-4196-s001.pdf]

Andrographolide impedes cancer stemness and enhances radio-sensitivity in oral carcinomas via miR-218 activation

Supplementary Materials

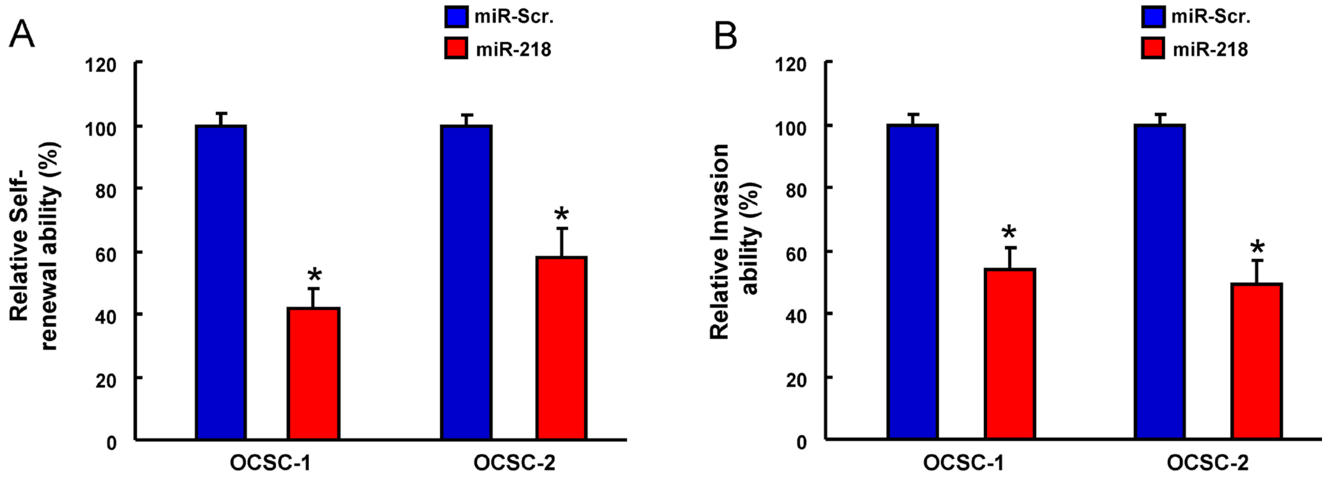

Supplementary Figure S1: miR-218 suppresses self-renewal and invasion abilities pLV-miR-218- and pLV-miR-Scr.-transfected OCSC cells were analyzed for secondary spheres-forming (**A**) and invasion (**B**) abilities. *$p < 0.05$ vs. Control.
